# Elevated Serum C-Reactive Protein Relates to Increased Cerebral Myoinositol Levels in Middle-Aged Adults

**DOI:** 10.1155/2012/120540

**Published:** 2012-02-22

**Authors:** Danielle E. Eagan, Mitzi M. Gonzales, Takashi Tarumi, Hirofumi Tanaka, Sandra Stautberg, Andreana P. Haley

**Affiliations:** ^1^Department of Psychology, The University of Texas at Austin, Austin, TX 78722, USA; ^2^Imaging Research Center, The University of Texas at Austin, Austin, TX 78759, USA; ^3^Department of Kinesiology and Health Education, The University of Texas at Austin, Austin, TX 78712, USA

## Abstract

C-reactive protein (CRP), a systemic marker of inflammation, is a risk factor for late life cognitive impairment and dementia, yet the mechanisms that link elevated CRP to cognitive decline are not fully understood. In this study we examined the relationship between CRP and markers of neuronal integrity and cerebral metabolism in middle-aged adults with intact cognitive function, using proton magnetic resonance spectrocospy. We hypothesized that increased levels of circulating CRP would correlate with changes in brain metabolites indicative of early brain vulnerability. Thirty-six individuals, aged 40 to 60, underwent neuropsychological assessment, a blood draw for CRP quantification, and ^1^H MRS examining N-acetyl-aspartate, myo-inositol, creatine, choline, and glutamate concentrations in occipito-parietal grey matter. Independent of age, sex and education, serum CRP was significantly related to higher cerebral myo-inositol/creatine ratio (*F*(4,31) = 4.74, *P* = 0.004), a relationship which remained unchanged after adjustment for cardiovascular risk (*F*(5,30) = 4.356, CRP *β* = 0.322, *P* = 0.045). Because these biomarkers are detectable in midlife they may serve as useful indicators of brain vulnerability during the preclinical period when mitigating intervention is still possible.

## 1. Introduction

Much evidence suggests that inflammatory conditions common in midlife such as obesity, atherosclerosis, and Type II diabetes play an important role in the development of dementia [1, 2]. Longitudinal studies have indicated that low-grade systemic inflammation in middle age adds to the risk of late-life cognitive impairment over and above the risk assessment afforded by disease (e.g., hypertension and atherosclerosis) and lifestyle habits (e.g., smoking) [1, 3, 4]. In addition, increases in serum inflammatory markers such as interleukin-6 (IL-6) and C-reactive protein (CRP) have been shown to exacerbate cognitive decline in older adults with metabolic syndrome, a condition defined by cooccurring obesity, hypertension, dyslipidemia, and hyperglycemia [2, 5]. The long-term use of anti-inflammatory drugs (NSAIDS), on the other hand, has been associated with a reduction in dementia risk [6–8], lending further support to the idea that sustained activation of an inflammatory immune response can foster neuronal vulnerability beyond what is expected of normal aging.

With increasing age, inflammatory changes are appreciable in the brain in the form of astrocyte proliferation and the presence of activated microglia [9]. However, it is still unclear in what capacity midlife inflammation initiates detectable changes in the brain that, if left unchecked, may progress or contribute to neurodegenerative conditions. Such information will be invaluable as chronic subclinical inflammation presents a potentially modifiable risk factor for future cognitive decline and dementia. Early detection of biomarkers linked to cognitive decline allows time for mitigating action to be taken, prior to the onset of irreversible clinical signs.

With this information as background, our goal was to investigate the relationship between systemic inflammation and cerebral metabolism in middle-aged adults with intact cognitive function. Specifically, we examined the association between serum levels of CRP and markers of neuronal integrity, osmolarity, and glial proliferation measured by magnetic resonance spectroscopy (^1^H MRS). To our knowledge, this is the first study to examine the relationship between an inflammatory marker and cerebral metabolites in cognitively healthy adults. As such, it offers novel information regarding the cooccurrence of increasing systemic inflammation and changes in neurochemistry within a middle-aged sample.

CRP is an acute phase protein released by hepatocytes in response to increases in circulating inflammatory cytokines [10]. CRP also serves as an activator of the complement system, an immunological cascade that assists the innate immune system by marking and destroying non-self antigens. Activation of complement is thought to contribute to the perpetuation of the inflammatory response and is implicated in neurodegenerative processes [11].

Perturbations in the levels of certain cerebral metabolites have been correlated with a variety of clinical conditions. Two markers in particular have been directly linked to changes in cognitive functioning: N-acetylaspartate (NAA) and Myoinositol (mI). NAA occurs exclusively in neurons and oligodendrocyte-type-2 astrocytes [12] and is widely regarded as a marker of neuronal viability, synaptic health, and metabolism [13]. Higher levels of NAA are linked to better cognitive test performance [14], and reduced levels are associated with cognitive impairment following brain injury [15] and neurodegenerative diseases [16]. Myoinositol (mI) is an organic osmolyte and purported glial marker [17]. Changes in levels of mI have been shown to presage the onset of cognitive decline in conditions fostering neuroinflammation, such as HIV and Alzheimer's disease [18–21]. Elevations of mI levels in Alzheimer's disease have been interpreted as a sign of gliosis [22].

We hypothesized that increased levels of circulating CRP would correlate with changes in brain metabolites indicative of early brain vulnerability, such as lower levels of NAA and/or higher levels of mI.

## 2. Materials and Methods

The study was approved by the Institutional Review Board of the University of Texas at Austin, and all volunteers provided written informed consent before enrollment. Participants were required to complete a medical history interview in which medical conditions and treatments were coded as either present or absent based on participants' self-report. Participants then underwent a full neuropsychological evaluation and a general health assessment, including a fasting blood draw for CRP. Visits were conduced on separate days and participants completed the study within one month.

### 2.1. Participants

 Participants between the ages of 40 and 60 were recruited through flyers and newspaper advertisements. Exclusion criteria included a history of neurological disease (e.g., large vessel stroke, seizure disorder, Parkinson's disease, clinically significant traumatic brain injury, multiple sclerosis, or brain infection/meningitis), major psychiatric illness (e.g., schizophrenia, bipolar disorder), substance abuse (diagnosed abuse and/or previous hospitalization for substance abuse), diabetes mellitus, serum CRP greater than 10 mg/L, or MRI contraindications. One hundred and three volunteers were screened; of these, 36 participants (14 men, 22 women) passed the initial screening process and were included in the final sample.

The mean (±SD) age of the sample was 49.7 ± 6.5 years. The mean education level was 15.0 ± 2.6 years. The mean full-scale IQ score was 112.3 ± 12.4, indicating high average global cognitive functioning according to published norms [23]. Enrollees identified themselves as follows: 42% Caucasian, 39% Hispanic, 3% Asian, 11% African American, and 6% Other.

### 2.2. Assessments

#### 2.2.1. General Health Screening

Fasting blood concentrations of glucose, triglyceride, HDL-cholesterol, and LDL-cholesterol were determined using the standard enzymatic technique. Arterial blood pressure was measured using a standard oscillometric blood pressure monitor (VP-2000, Colin Medical Instruments, San Antonio, TX) after at least 15 minutes of rest. Body Mass Index (BMI) was calculated as weight in kilograms divided by the square of height in meters. Since CRP is also a recognized cardiovascular risk factor [24], and cardiovascular disease is known to impact cognition [2, 25, 26], cardiovascular disease (CVD) risk factors (including obesity, hypertension, dyslipidemia, hyperglycemia, and history of smoking) were coded as present or absent (1 or 0) based on the American Heart Association recommendations for risk criteria [27]. These risk factors were then summed to create an overall CVD risk score (range 0–5) that was included as a covariate in subsequent analyses.

#### 2.2.2. High Sensitivity C-Reactive Protein (CRP) Assay

A 3 mL fasting blood sample was collected from the antecubital vein by venipuncture. Serum was separated within 2 hours of the collection, and aliquots were stored at −80°C until later analysis. Prior to the analysis, the serum sample was diluted 100-fold. Serum concentration of CRP was measured using high sensitivity human ELISA kits (Alpha Diagnostics, San Antonio, TX) with a minimum detectable concentration of 0.35 ng/mL.

#### 2.2.3. Neuropsychological Evaluation

All participants completed a two-hour assessment battery including standard clinical neuropsychological instruments with established reliability and validity [28]. Neuropsychological measures were grouped into one of five cognitive domains: (1) global cognitive functioning, (2) language functions, (3) memory functions (4) attention-executive-psychomotor functions, and (5) visual-spatial abilities. The following test scores were included in each domain, and raw total scores were utilized: (1) global: WASI Full Scale IQ [23] and Mini-Mental State Examination (MMSE) [29]; (2) language: WASI Vocabulary Subtest and Category Fluency for Animals [30]; (3) memory: CVLT-II immediate recall, delayed recall, recognition discrimination [31], Rey Complex Figure Test (RCF) immediate recall, delayed recall, and recognition discrimination [28]; (4) attention-executive-psychomotor functioning: WAIS-III, Digit Span Subtest [32], Controlled Oral Word Association Test, (COWAT) [33], Trail Making Test A and B [34], and Grooved Pegboard-Dominant Hand time to completion [35]; (5) visual-spatial abilities: RCF copy and WASI Matrix Reasoning Subtest. Emotional functioning was also assessed with the Beck Depression Inventory-II (BDI) [36] and the Trait Anxiety Inventory (STAI-T) [37]. All tests were administered and scored by a trained research assistant using standard administration and scoring criteria. Participants' raw test scores were converted to z-scores using the study sample mean and standard deviation. Timed test scores were multiplied by −1 so that higher scores indicate better performance. Five composite cognitive domain z-scores were calculated for each participant by averaging the z-scores of all tests within that domain.

#### 2.2.4. Neuroimaging

**Figure 1:**
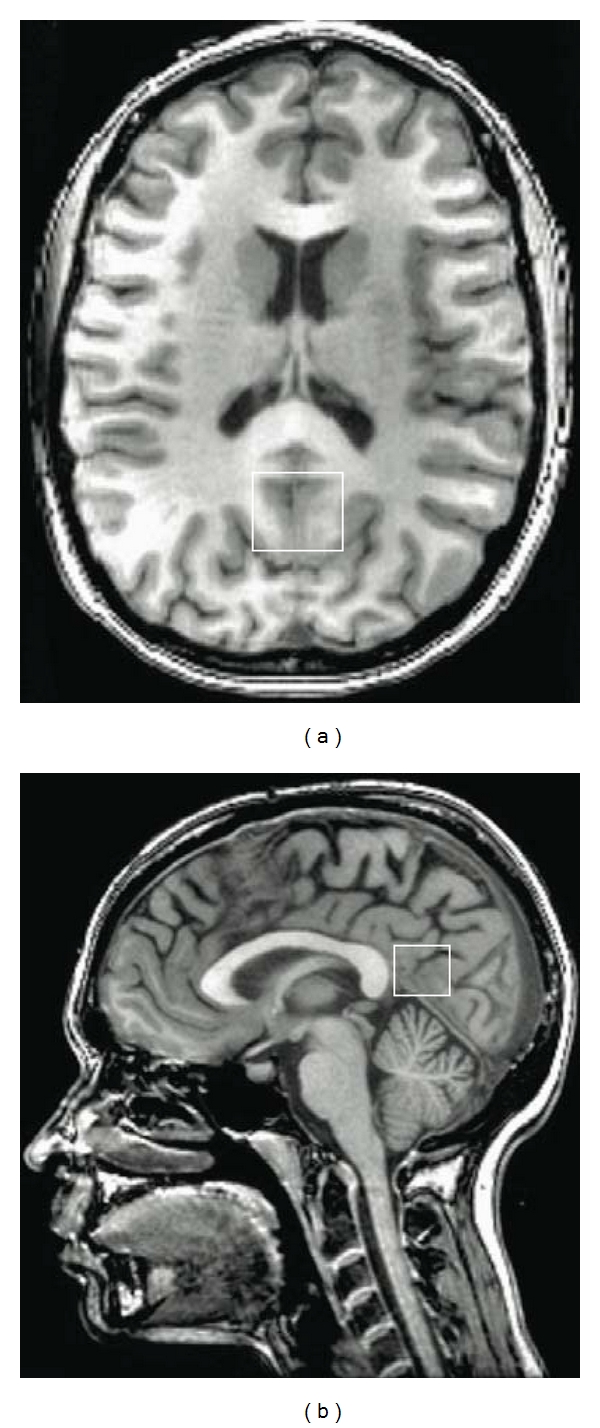
Anatomic images with superimposed voxel borders indicating MRS volume in occipitoparietal grey matter.

 Structural imaging included a high-resolution Spoiled Gradient Echo (SPGR) sequence (256 × 256 matrix, FOV = 24 × 24 cm^2^, 1 mm slice thickness, 0 gap) anatomical scan of the entire brain in the sagittal plane. ^1^H MRS data for each participant were acquired in a single session on a 3T GE Signa Excite MRI scanner equipped with a standard head coil. Single voxel proton ^1^H MRS was performed using the automated GE pulse sequence PROBE-P, which is a point resolved spectroscopy (PRESS) sequence with chemical shift selected (CHESS) water suppression. ^1^H-MRS parameters were as follows: echo time/repetition time (TE/TR) = 35/3000 ms, 128 excitations, 5000 Hz spectral width, volume ~6 cm^3^ from the occipitoparietal gray matter (Figure 1). The region was chosen because spectroscopically detectable alterations in its neurochemical composition are well documented in disorders of cognition, in correspondence with severity of cognitive dysfunction [22]. Commercially available software, LCModel, was used to quantify and separate the metabolite resonances from the macromolecule background [38]. In line with standard clinical protocols, the concentrations of NAA, choline containing compounds (choline, phosphocholine and glycerophosphocholine, Cho), myoinositol, and glutamate (Glu) were reported as ratios relative to creatine (Cr) [22, 39]. MRS data for all participants in the final sample met quality control criteria (Cramer-Rao Lower Bounds for NAA, mI, Cho or Glu <12).

 Within pathological conditions, the use of Cr as a stable standard for comparison of other metabolites is debated, as are the benefits of reporting absolute versus fractional quantities of metabolites. We opted to report metabolite concentrations in ratio to Cr because our sample consists of healthy middle-aged individuals, and because ratios are more easily obtained during clinical scans and hence possess more clinical utility. 

### 2.3. Data Analyses

The relationship between CRP and the ^1^H MRS markers (NAA/Cr, Glu/Cr, Cho/Cr and mI/Cr) was analyzed using a single multivariate multiple linear regression model with all MRS parameters entered in at once. Age, sex, and level of education were chosen as covariates *a priori,* since they are known to influence the risk of cognitive vulnerability [40, 41]. Follow-up analyses of the relationship between mI/Cr and CRP were conducted using linear regression. All statistical analyses were performed using SPSS 16.0 (SPSS Inc., Chicago, IL). A two-tailed-alpha level of 0.05 was used as the criterion for statistical significance.

## 3. Results

### 3.1. Selected Subject Characteristics

Selected characteristics of the subjects are reported in Table 1. Raw cognitive test scores and standard deviations are reported in Table 2. Descriptive statistical analyses revealed a cognitively normal, ethnically diverse, and middle-aged sample, well representative of the population of the state of Texas based on 2000 US census data for the state. Regression residuals were normally distributed according to the Shapiro-Wilk test of normality (Shapiro-Wilk > 0.95, *P* > 0.95), and no transformations were performed.

### 3.2. Demographics and Cardiovascular Risk in relation to CRP and Cerebral Metabolism

CRP was not significantly related to age, sex, or education level. As expected, increased CRP was significantly related to CVD risk (*r* = 0.346, *P* = 0.039). We quantified CVD risk as a 0–5 index score based on the current presence of each of the following risk factors: obesity, hypertension, dyslipidemia, hyperglycemia, and history of smoking. Among cerebral metabolites, male sex was correlated with higher mI/Cr (*r* = 0.44, *P* = 0.007). No other cerebral metabolites reached statistical significance in relation to demographic variables. Similarly, no cerebral metabolites were significant relative to level of CVD risk. Among the variables used to assess CVD risk, obesity and elevated fasting glucose were each significantly associated with higher CRP (*β* = 2.310, *t* = 2.302, *P* = 0.028 and *β* = 2.396, *t* = 2.091, *P* = 0.045, resp.), whereas a history of smoking (*β* = −0.472, *t* = 0.454, *P* = 0.653), dyslipidemia (*β* = −0.026, *t* = −0.025, *P* = 0.981), and hypertension (*β* = −1.416, *t* = −1.453, *P* = 0.157) were not. 

### 3.3. Cognition in relation to CRP and Cerebral Metabolism

We found no significant relations between CRP and measures of global cognitive functioning (*r* = −0.15, *P* = 0.92), language (*r* = −0.12, *P* = 0.77), memory (*r* = −0.22, *P* = 0.37), attention-executive-psychomotor functioning (*r* = −0.13, *P* = 0.35), or visual-spatial ability (*r* = −0.13, *P* = 0.90). Nor did we find significant relations between NAA/Cr, Cho/Cr, Glu/Cr, or mI/Cr and any domain of cognitive functioning. These findings were not surprising considering our relatively young, cognitively intact sample (average global cognitive function (FSIQ) = 112.3).

### 3.4. CRP in relation to Cerebral Metabolism

Relationships between CRP and cerebral metabolism were examined by multivariate multiple regression analyses adjusted for sex, age, and years of education. In fully adjusted analyses, CRP was positively associated with cerebral mI/Cr in the brain (*F*(4,31) = 4.74, *P* = 0.004) but not with NAA/Cr (*F*(4,31) = 0.99, *P* = 0.427), Cho/Cr (*F*(4,31) = 0.53, *P* = 0.715) or Glu/Cr (*F*(4,31) = 1.64, *P* = 0.188). Coefficients are reported in Table 3. This relationship remained unchanged even after adjustment for cardiovascular risk (*F*(5,30) = 4.356, CRP *β* = 0.322, *P* = 0.045).

## 4. Discussion

 We found that higher serum CRP, a sensitive marker of systemic inflammation, was significantly related to higher cerebral mI/Cr concentrations in cognitively normal middle-aged adults. This finding adds to the growing body of literature relating CRP to neurochemical changes linked to the development of cognitive impairment in later life [1, 25, 42–44]. It extends the previous literature by suggesting that systemic inflammation is related to neurochemical changes even in middle-aged adults with intact cognitive functioning.

We chose to examine CRP because it presents a stable, easily obtained inflammatory marker that is not subject to variation based on time of sampling or blood glucose levels [45]. CRP is also a well-known risk factor for vascular disease and endothelial dysfunction [27, 46–49], both of which contribute to the rate and severity of cognitive decline over the lifespan [50, 51]. Perturbations in cerebral mI/Cr, an organic osmolyte and marker of glial proliferation [17], have also been shown to precede the onset of cognitive decline in conditions fostering immune activation and neuroinflammation [18–21]. However, until now, no studies have specifically looked for a correlation between cerebral mI/Cr levels and systemic inflammation. Our results indicate that increased systemic inflammation covaries with increased cerebral mI/Cr. While our study does not allow for inferences regarding a mechanistic linkage between CRP and changes in mI/Cr, it nonetheless highlights a potential relationship between these two factors. Importantly, this relationship is detectable in midlife, prior to clinically significant changes in cognitive status.

One explanation for the increased mI/Cr observed in our sample could be increased brain water diffusion in the presence of proinflammatory conditions. Because of its degrading effect on tissue integrity, inflammation has been positively correlated with increased white matter diffusion in neurodegenerative disease [52, 53], acute neuroinflammatory conditions [54], and normal aging [55]. In turn, increased brain water diffusion has been linked to higher levels of cerebral osmolytes including mI/Cr [20]. Systemic inflammation and resultant endothelial dysfunction could also contribute to proinflammatory conditions in the brain [2, 47], leading to increased brain water diffusion and glial accumulation of osmolytes such as mI/Cr [44]. CRP in particular has been implicated in autotoxic cascades leading to decreased endothelial integrity [56], which over time may impair cerebrovascular autoregulation and negatively impact cognition [57].

Although our sample consisted of healthy adults without clinical signs of neuroinflammation, many of them were positive for one or more cardiovascular risk factors (e.g., hypertension, obesity) associated with increased circulating inflammatory markers and endothelial dysfunction. In particular, the mean BMI of our sample, 29.7 (SD = 5.6), is considered obese according to clinical guidelines defining obesity as a BMI equal to or greater than 30 kg/m^2^ [58]. Abdominal obesity has been linked to elevated levels of CRP [59] and is understood as a causative factor for endothelial dysfunction [60]. It is possible that the higher mean BMI observed in our sample is one factor contributing to endothelial dysfunction and accompanying changes in the neurochemical milieu.

Additionally, the mean CRP value of our sample (2.72 mg/L) falls just below the high-risk category for cardiovascular disease events according to the American Heart Association [27]. The American Heart Association's CRP risk categories (<1.0 mg/L = low, 1.0 to 3.0 mg/L = average, and >3.0 mg/L = high) are based on CRP tertiles observed in the adult population. The high-risk tertile has roughly twice the relative risk of a cardiovascular disease event compared to the low-risk tertile [27]. The mean CRP value obtained for our participants, especially taken together with the high mean BMI, indicates that our sample may be at greater risk for decreased endothelial integrity and increased brain water diffusion. However, it should be stressed again that the correlation between elevated CRP and elevated mI/Cr does not indicate a mechanistic linkage between the two, and the explanations offered for elevated mI/Cr in this sample remain hypothetical.

A related explanation for increased mI/Cr in our sample is increased blood brain barrier (BBB) permeability [61], which may contribute to neuronal vulnerability and eventual cognitive decline. Myoinositol accumulates preferentially in astrocytes, which, together with endothelial tight junctions, provide selective permeability of the BBB. BBB permeability increases in the presence of inflammatory markers and may lead to toxic elevations of intracellular calcium [62]. In our sample increased levels of mI/Cr could be interpreted as an early sign of astrocyte proliferation related to impaired endothelial function and resultant osmotic stress in response to low-grade systemic inflammation. Support for this hypothesis comes from the upregulation of mI synthesis in the presence of conditions causing BBB permeability, such as trauma, carbon monoxide poisoning, demyelination, beta-amyloid deposits [63], and neurofibrillary tangles [64]. These conditions represent extreme events leading to neuronal vulnerability and cell death and are not assumed to be present in our sample. However, taken together, our observed increases in mI/Cr and CRP could be viewed as two early indicators of neuronal vulnerability similar to though less extreme than the conditions above. Further support for this hypothesis comes from evidence that elevated mI/Cr precedes changes in the levels of other cerebral metabolites in diseases marked by cognitive decline, such as Alzheimer's disease and other dementias [18].

 The primary strength of our study was the thorough characterization of our sample in terms of cognitive and physiological functioning. The cognitive battery utilized in the study examined multiple domains of cognitive functioning and relied on at least two measures within each domain. Assessments of metabolic and vascular health were performed by trained researchers rather than relying on self-reported medical history, which may contain unintentional omissions and errors. External confirmation of cognitive and physiological health status has allowed us to interpret our findings with greater confidence, without the potentially confounding effects of underreported medical conditions or cognitive disorders.

 The cross-sectional design of our study prevents determination of a causal link between inflammation and increased mI/Cr and demonstrates only that there may be a relationship between these two factors. It is highly likely that multiple factors in addition to these contribute to cognitive decline over the lifespan. Our small, well-educated sample also limits the generalizability of our findings. Moreover, we did not obtain any additional assessments of neuronal integrity, such as Diffusion Tensor Imaging, white matter hyperintensities, or additional markers of inflammation, such as IL-6. Future longitudinal studies beginning in midlife and conducted with larger, randomly selected community samples could help elucidate potential interactions between systemic inflammation and neural metabolism and the extent to which these factors predict cognitive decline. Additionally, combining multiple measures of neuronal health and multiple measures of inflammation would offer a more complete picture of the ways in which brain health is affected by systemic inflammation. Finally, we did not quantify other conditions known to correlate with CRP levels, such as alcohol consumption, stress, and sleep quality. Future studies should further examine these variables as they relate to CRP levels and changes in cerebral mI/Cr.

## Figures and Tables

**Table 1:** Selected characteristics of the subjects.

		Mean (SD)	%	Median	Range
Gender					
14 men					
22 women					
Age		49.7 (6.5) years			
Education level		15.0 (2.6) years			
Caucasian			42		
Hispanic			39		
African American			11		
Other			6		
Asian			3		
BMI (kg/m^2^)		29.7 (5.6)		29.29	21–56
CRP (mg/L)		2.72 (2.8)		1.32	.0006–9.53
Hypertension			30.5	125/75	104/61–183/98
High LDL			55.5	108	42–236
Hyperglycemia			13.9	97	80–276
Smokers			5.6		

**Table 2:** Cognitive characteristics of the subjects.

Test measures by domain	Sample mean score (SD)	Total possible score
Global cognitive functioning		
Mini-Mental Status Exam (MMSE)	28.4 (1.3)	30
Wechsler Abbreviated Scale of Intelligence (WASI)	112.3 (12.4)	95% is 149–160
Language		
WASI Vocabulary Subtest	62.4 (10.4)	80
Category Fluency for Animals (Animals)	23.8 (5.5)	1-minute limit
Visual-spatial		
Complex Figure Test (CFT-Copy)	30.9 (4.2)	36
WASI Matrix Reasoning Subtest	25.9 (4.9)	35 (12–44 yrs) or 32 (45–79 yrs)
Memory		
California Verbal Learning Test (CVLT)		
Immediate recall	5.4 (1.8)	16
Delayed recall	11.0 (3.2)	16
Recognition (Yes/No)	3.1 (0.8)	−4 to 4
Complex Figure Test (CFT)		
Immediate recall	15.9 (5.3)	36
Delayed recall	15.4 (5.5)	36
Recognition discrimination	18.7 (4.1)	24
Attention-executive-psychomotor		
Trail Making Test A, time in seconds (Trails A)	29.6 (8.9)	5-minute limit
Trail Making Test B, time in seconds (Trails B)	74.8 (28.9)	5-minute limit
Controlled Oral Word Association Test (COWAT)	36.4 (9.8)	3-minute limit
Grooved Pegboard, Dominant Hand, time in seconds (Pegs-D)	76.6 (14.8)	No limit
WAIS-III Digit Span Subtest (Digit Span)	16.9 (4.2)	30

**Table 3:** Multiple regression analyses depicting independent correlates of Ins/Cr, NAA/Cr, Glu/Cr, and Cho/Cr.

Model	*R* ^2^ (*P* value)	Variable	*B*	*β*	*P* value	95% CI Lower bound	95% CI Upper bound
*Dependent variable: Ins/Cr*							

(1)	0.379 (0.004)	Sex	0.104	0.539	0.001	0.046	0.161
		Age	−0.002	−0.102	0.480	−0.006	0.003
		Years of education	0.011	0.301	0.054	0.000	0.023
		CRP	0.013	0.375	0.015	0.003	0.023

*Dependent variable: NAA/Cr*							

(2)	0.113 (0.427)	Sex	0.021	0.107	0.545	−0.050	0.092
		Age	0.005	0.293	0.097	−0.001	0.010
		Years of education	−0.005	−0.134	0.461	−0.019	0.009
		CRP	0.000	0.009	0.958	−0.012	0.013

*Dependent variable: Glu/Cr*							

(3)	0.175 (0.188)	Sex	0.067	0.209	0.226	−0.044	0.178
		Age	−0.005	−0.201	0.233	−0.014	0.004
		Years of education	0.024	0.394	0.030	0.003	0.046
		CRP	0.001	0.011	0.947	−0.019	0.020

*Dependent variable: Cho/Cr*							

(4)	0.064 (0.715)	Sex	0.010	0.212	0.247	−0.007	0.028
		Age	0.000	0.067	0.706	−0.001	0.002
		Years of education	−0.001	−0.080	0.666	−0.004	0.003
		CRP	0.000	0.025	0.890	−0.003	0.003

## Abbreviations

CRP: C-reactive protein
NAA: N-acetyl aspartate
mI: myo-inositol
mI/Cr: the ratio of myo-inositol to creatine.
